# Preferences of Canadian Patients and Physicians for Treatment of HR+/HER2− Advanced Breast Cancer

**DOI:** 10.3390/curroncol28010051

**Published:** 2021-01-14

**Authors:** Daniel Stellato, Marroon Thabane, Caitlin Eichten, Thomas E. Delea

**Affiliations:** 1 Policy Analysis Inc., Chestnut Hill, MA 02467, USA; dstellato@pai2.com (D.S.); ceichten@pai2.com (C.E.); 2 Novartis Pharmaceuticals Canada Inc., Dorval, QC H9S 1A9, Canada; marroon.thabane@novartis.com

**Keywords:** advanced breast cancer, preferences

## Abstract

(1) Background: Past research suggests that patients with advanced breast cancer prefer treatments with improved clinical outcomes and lower risk of side effects. Evidence on preferences of Canadian patients and physicians for treatments for advanced breast cancer is limited. (2) Methods: Patients’ and physicians’ preferences for treatments for HR+/HER2−, pre-/peri-menopausal advanced breast cancer were assessed by an online discrete choice experiment (DCE). Treatment alternatives were characterized by seven attributes regarding dosing, efficacy, and toxicities, with levels corresponding to those for ribociclib plus a non-steroidal aromatase inhibitor (NSAI), NSAI, and tamoxifen. For patients, impacts of advanced breast cancer on quality of life (QOL) and ability to work/perform activities of daily living also were assessed. Patients were recruited by a Canadian breast cancer patient advocacy group through email and social media. Physicians were recruited by email. (3) Results: Among 118 patients starting the survey, 23 completed ≥ 1 DCE question (19%). Among 271 physicians who were sent the e-mail invitation, 21 completed ≥ 1 DCE question (8%). For both patients and physicians, the increased probability of remaining alive and without cancer progression over 2 years was the most important attribute. A treatment with attributes consistent with ribociclib plus NSAI was chosen by patients and physicians in 70% and 88% of the time, respectively. A substantial proportion of patients reported worrying about future diagnostic tests and their cancer getting worse; (4) Conclusions: Canadian patients and physicians are generally concordant in preference for advanced breast cancer treatments, preferring ribociclib plus NSAI to other options.

## 1. Introduction

Cancer is a prominent cause of death among women, second only to heart disease [1]. Breast cancer is the second leading cause of cancer deaths among women in both Canada and the US [2,3]. It is estimated that in 2017 in Canada, 26,300 women were diagnosed with breast cancer, making it the most commonly diagnosed cancer and accounting for 25% of all incident cases in women [3]. It also is estimated that 5000 Canadian women died from breast cancer, which represents 13% of cancer deaths among women in 2017 [3]. Based on data collected by the Canadian Cancer Society (2006–2008), the five-year survival rate for breast cancer is estimated to be 79% in men and 87% in women [3]. Approximately 17% of breast cancer diagnoses in Canada are among women aged < 50 years; however, most clinical trials of breast cancer are in postmenopausal women only [4]. The majority of breast cancers cases are positive for the hormones estrogen and progesterone (HR+) and negative for human epidermal growth factor-2 (HER2−). These types of breast cancers account for approximately 72.7% of all breast cancer—64.8% of cases in women aged ≤ 50 years [5]. Advanced breast cancer (ABC) includes Stage III disease with inoperable tumors (i.e., “locally advanced”) and Stage IV disease (i.e., “metastatic”). Among women with newly diagnosed breast cancer in Canada between 2011 and 2015, 12.4% of cases were Stage III and 4.9% were Stage IV [6].

Median overall survival (OS) for ABC is approximately 2–3 years, and despite improvements in outcomes over the past decade, estimates of 5-year survival remain discouraging—approximately 25% [7,8]. The principal goals of treatment of ABC are the prolongation of life and at least maintenance of quality of life (QOL). Patients with ABC, whether pre-menopausal (preM), peri-menopausal (periM) or postmenopausal (postM) are usually treated with endocrine (i.e., hormonal) therapies (ETs) if HR+, with or without targeted therapies, or separately with chemotherapy (CT).

Treatment guidelines published by the European Society for Medical Oncology (ESMO), National Comprehensive Cancer Network (NCCN), and Canadian oncologists support the use of ET with or without cyclin-dependent kinase (CDK) 4/6 as first-line treat for the majority of patients with HR+ ABC [9,10,11]. CT may also be given as first-line treatment, but it is only recommended for patients with visceral crisis [9,11]. Despite the continuing development and availability of therapeutic options, many HR+/HER2− patients do not respond to their initial treatment, and the remainder will relapse eventually [12].

For pre-/peri-menopausal patients, there is consensus among the American Society of Clinical Oncology (ASCO), NCCN, and ESMO that the preferred first-line treatment should also include ovarian suppression or ablation in combination with hormone therapy [10,13,14]. Similar outcomes in ABC are reported for ovarian suppression with use of luteinizing hormone-releasing hormone (LHRH) agonists (e.g., goserelin) or ablation with oophorectomy [14] Pre- and peri-menopausal patients have not typically been included in clinical trials of first-line hormonal therapy, and few studies have been designed exclusively for such patients [14].

MONALEESA-7 (NCT02278120) is a phase 3, randomized, double-blind, placebo-controlled study of ribociclib in combination with a non-steroidal aromatase inhibitor (NSAI) and goserelin or tamoxifen and goserelin in pre- and peri-menopausal women with HER2−/HR+ ABC not amenable to curative therapy who had not received prior treatment for ABC (except ≤ 14 days of tamoxifen or NSAI ± goserelin for ABC) [15]. Between 17 December 2014 and 1 August 2016, 672 patients were randomly assigned to either ribociclib (*n* = 335) or placebo (*n* = 337) [16]. Based on the preliminary analysis with data cut-off of 20 August 2017, median progression-free survival (PFS) was 23.8 months (95% confidence interval [CI] 19.2–not reached) in the ribociclib group compared with 13.0 months (11.0–16.4) in the placebo group (hazard ratio (HR) 0.55, 95% CI 0.44–0.69; *p* < 0.0001) [16]. In the subgroup of patients receiving NSAI, median PFS was 27.5 months (19.1—not reached) for ribociclib compared with 13.8 months (12.6–17.4) for placebo (HR 0.57, 95% CI 0.44–0.74) [16]. An updated analysis with data cut-off of 30 November 2018 demonstrated a statistically significant improvement in OS for the ribociclib group compared with the placebo group (median not reached vs. 40.9 months, respectively; HR 0.71, 95% CI 0.54–0.95, log-rank *p* = 0.00973) [17]. Similar findings were reported for the subgroup of patients receiving an NSAI (HR 0.70, 95% CI 0.50–0.98) [17]. Grade 3 or 4 adverse events (AEs) reported in more than 10% of patients in either group were neutropenia (203 [61%] of 335 patients in the ribociclib group and 12 [4%] of 337 in the placebo group) and leucopenia (48 [14%] and four [1%]) [16]. Based on these results, the authors concluded that ribociclib plus endocrine therapy improved PFS compared with placebo plus ET and had a manageable safety profile in patients with premenopausal, HR+/HER2− ABC and that the combination of ribociclib plus endocrine therapy could represent a new first-line treatment option for these patients [16].

In light of the differences in efficacy, toxicity, and frequency and mode of administration among existing and novel treatments, patients and physicians need to consider trade-offs of benefits and risks when choosing among treatment options. At the time this study was conducted, robust data on OS from MONALEESA-7 were not available. Past research suggests that ABC patients preferred treatments with improved clinical outcomes and lower risk of side effects [18]. However, there is limited evidence on the preferences of Canadian patients with ABC or physicians who treat such patients regarding which treatment they might prefer among existing and novel treatments, in particular the CDK4/6 inhibitor ribociclib in combination with an NSAI and an LHRH agonist in pre-/peri-menopausal women.

## 2. Experimental Section

### 2.1. Overview

This was a descriptive, cross-sectional, online survey of Canadian patients with metastatic or ABC and physicians who treat breast cancer patients. The primary analysis focused on pre-/peri-menopausal patients with HR+/HER2− disease, and a secondary analysis was conducted based on all ABC patients regardless of menopausal or HR/HER2 status. The survey was conducted from June 2019 to July 2019. Patient participants were recruited in collaboration with the Canadian Breast Cancer Network (CBCN)—a patient advocacy group—through emails (“e-Blast”), posting to CBCN’s websites, and posting to social media (Twitter^®^ and Facebook^®^). Physicians were recruited through email lists provided by the study sponsor. The eligibility of all participants was assessed via a self-completed online screener.

Patient and physician preferences and relative importance for attributes of treatments for pre-/peri-menopausal, HR+/HER2− ABC were assessed by discrete choice experiment (DCE). DCE is a quantitative method that can be used to assess preferences for attributes associated with different treatment alternatives [19]. DCE involves providing individuals with a series of choice tasks wherein the individuals must choose between hypothetical treatment alternatives that differ in terms of levels of attributes of interest, with the levels of attributes systematically varied across respondents. Then, responses to the survey are used to estimate the degree to which each attributes influence preferences and to derive utility weights for each attribute level [20]. The attributes and their levels were selected to reflect differences in key characteristics of potential treatments for pre-/peri-menopausal patients with HR+/HER2− ABC, including ribociclib plus an NSAI plus an LHRH agonist (ribociclib + NSAI), an NSAI plus an LHRH agonist (NSAI), and tamoxifen plus an LHRH agonist (TAM) (Since all the treatment alternatives include an LHRH, for the remainder of the report, we do not reference this component of treatment).

The DCE used in this study employed a choice-based conjoint (CBC) approach with identical discrete choice tasks for the patient and physician surveys. For each discrete choice task, participants were presented with a series of choices among two hypothetical treatment alternatives. Three fixed choice tasks were also included in the survey in which participants would choose between attribute profiles corresponding to actual treatments (i.e., ribociclib + NSAI, NSAI, and TAM). Responses to the fixed tasks were used to estimate proportions of patients and physicians who would prefer one treatment over another (respondents were not able to discern whether the attribute profiles corresponded to any actual treatment in the fixed tasks). For patients, the survey also included a number of questions concerning patients’ demographic and clinical characteristics, ABC treatment history, work productivity, fertility and reproduction concerns, and psychological well-being. The DCE was developed in a manner consistent with the published recommendations [19,21,22]. The survey was self-completed and administered and analyzed using Sawtooth Software (Sun Valley, ID, USA), which is an online survey software and conjoint analysis tool [23]. The study received Institutional Review Board approval by Advarra: Research Compliance Solutions (Columbia, MD, USA; Aurora, ON, Canada).

### 2.2. Survey Participants

Patient eligibility criteria for the primary analysis included residence in Canada, female aged 18 to 59 years, current diagnosis of metastatic or ABC, HR+/HER2− breast cancer, and pre- or peri-menopausal or with medically- or surgically-induced menopause. These criteria were selected to match the population of patients who would, currently, or at some future point be eligible for treatment with ribociclib + NSAI based on inclusion criteria of MONALEESA-7. Approximately two weeks after the first participant had enrolled in the study, it was determined that the sample size for the patient sample would likely be inadequate. Accordingly, the eligibility criteria were amended so that all adult women with ABC, regardless of HR/HER2 or menopausal status would be eligible. Therefore, the primary analysis was defined as pre- or peri-menopausal women with HR+/HER2− ABC (i.e., consistent with the original criteria prior to the amendment) while a secondary analysis included all patients who enrolled in the study before or after the amendment. Physicians were eligible who were aged at least 18 years, held a license to practice medicine in Canada, had specialization in medical oncology, and reported seeing at least one ABC patient in the past year.

### 2.3. Survey Questionnaire

Attributes and levels employed in the DCE were determined based on consultation with clinical experts and representatives of patient groups and were designed to capture key differences between three treatments of interest for pre- and peri-menopausal women with HR+/HER2− ABC, which included ribociclib plus NSAI, NSAI, and TAM. Seven attributes were evaluated, including dosing schedule, follow-up and monitoring requirements, estimated PFS, improvement in pain, and chance of hot flashes, neutropenia, and nausea (Table 1). While OS is regarded as the gold standard of evidence for the efficacy of oncology therapies, such data from clinical trials are often incomplete due to improvements in outcomes with novel therapies and length of follow-up at the time of the preliminary analysis [24]. A recent study by Forsythe and colleagues demonstrated a statistically significant correlation between median PFS and OS in HR+/HER2− ABC, which suggests that PFS may be an appropriate surrogate for OS [25]. Given that data on OS from MONALEESA-7 were immature at the time the study was conducted, OS was not included as an attribute. Follow-up and monitoring requirements, PFS, and chance of hot flashes had three levels, with one level corresponding to each of the three treatments of interest. Dosing schedule, improvement in pain, chance of neutropenia, and chance of nausea had two levels, as it was assumed these attributes were identical for patients receiving an NSAI or TAM. Attribute profiles corresponding to treatment with ribociclib + NSAI, NSAI, and TAM are presented in Table 2.

The dosing schedules for treatments of interest were based on the published report of the MONALEESA-7 trial [16]. Levels related to landmark PFS were based on subgroup data from the MONALEESA-7 trial (data on file). While data on PFS for MONALEESA-7 are reported by treatment group [16], there was no published report of PFS by the endocrine-partner subgroup at the time this study was conducted. Descriptions of attributes for AEs were based on Common Terminology Criteria for Adverse Events v5.0 for grade 2 AEs [26]. The levels for the probabilities of AEs were based on the overall incidence of these events (regardless of grade) in key clinical trials of the treatments of interest as reported in product monographs and trial reports [27,28,29]. Frequency of healthcare services for follow-up and monitoring were based on product monographs for the treatments of interest [30,31,32]. Levels for improvement in amount of pain experienced were based on analyses of the European Organization for Research and Treatment of Cancer Quality of Life Questionnaire (EORTC QLQ-C30) pain score in MONALEESA-7, assuming effects on pain scores would be the same for patients receiving an NSAI or TAM [16].

The experimental design of the DCE was generated to conform to principles of minimal overlap (i.e., levels that appear multiple times in the same task), level balance (i.e., each level appears with approximately the same frequency), and orthogonality (i.e., the weight of each attribute level can be measured independently of all other attribute levels) using Sawtooth Software (Sun Valley, ID, USA). The survey was comprised of 13 tasks based on the experimental design and three fixed, head-to-head tasks comparing profiles corresponding to ribociclib + NSAI versus NSAI; ribociclib + NSAI versus TAM; and NSAI versus TAM. For the 13 tasks based on the experimental design, the attribute levels were varied systematically so that each respondent answered a different set of tasks. Treatment labels were omitted from all tasks so that respondents would not be able to discern whether a given concept within a task corresponded to a real or hypothetical treatment.

The patient survey included secondary endpoints related to patients’ anxiety and depression [33,34], perceived cancer control [35], fear of cancer progression [36], fertility and reproduction concerns [37], and work productivity and impairment [38] in addition to the DCE. A copy of the survey instrument is included in the Supplementary Materials.

### 2.4. Survey Pre-Test

A pre-test of ten patients and two physicians was conducted to ascertain the time required to complete the survey and whether patient and physician participants found the instructions for completing the survey and the descriptions of the characteristics of the treatments easy to understand. Among the ten patients who started the pre-test, four met eligibility criteria for the primary analysis, three consented to participate in the survey, and two completed the choice tasks; both physicians who started the pre-test met eligibility criteria, consented, and completed the choice tasks. Based on the pre-test, minor changes were made to the descriptions of eligibility criteria included in the study screener questions. Since there were no material changes to the survey based on the pre-test, the responses from the pre-test phase were included in the analysis along with those based on the final survey instrument.

### 2.5. Analyses

Descriptive statistics were generated to summarize characteristics of physician and patient participants, patients’ and physicians’ relative preferences for key treatment attributes and for treatment options with profiles corresponding to ribociclib + NSAI, an NSAI, and TAM. For patients, descriptive statistics also were generated to summarize levels of anxiety and depression, perceived cancer control, fear of cancer progression, work productivity and activity impairment, and concerns regarding fertility/reproduction.

Estimates of relative preferences for attributes of treatments for ABC were estimated separately for patients and physicians using hierarchical Bayes (HB) approach and assuming preferences were normally distributed across respondents and effects-coded variables [22]. The HB method provides estimates of individual part-worth utilities for each respondent, which are then used to construct the joint posterior distribution of preference weights for all respondents. The estimated preference weights (i.e., utilities) from the HB analysis were used to calculate the conditional relative importance of the attributes, which indicate how much weight respondents place on each attribute when deciding between treatments [39]. The relative importance for an attribute is estimated by taking the difference in preference weights between the most preferred level of an attribute and the least preferred level of that attribute.

## 3. Results

### 3.1. Study Participants

**Figure 1:**
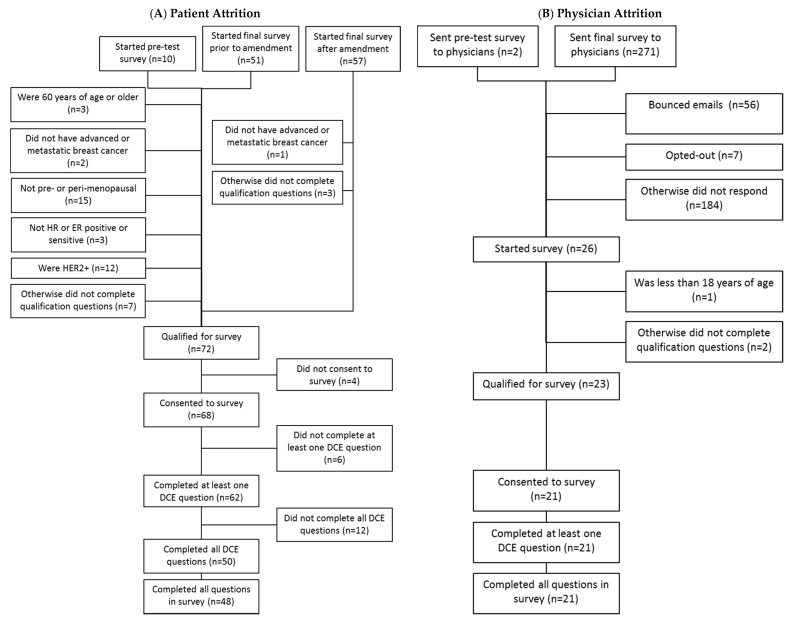
Patient and physician attrition; Note: Numbers in parentheses are numbers of subjects.

A total of 118 patients started the survey, including 10 who started the pretest, 51 who started the final survey prior to the amendment, and 57 who started after the amendment. Of these, 27 met eligibility criteria for the primary analysis, including 23 who consented to the survey and completed at least one discrete choice task, and 17 who completed all questions in the survey. Among all patients, 72 qualified for the survey, 68 consented to the survey, 62 completed at least one DCE question, and 48 completed all survey questions. A total of 271 physicians were sent the e-mail invitation to participate in the survey, and two physicians were sent the pre-test survey. Of these, 26 started the survey, 23 qualified for the survey, 21 consented to the survey, and 21 completed at least one DCE question. All the physicians who started the DCE completed all the DCE tasks. Figure 1 summarizes the patient and physician attrition for the survey.

### 3.2. Participants Characteristics

Patient and physician characteristics are reported in Table 3. Mean age was 46 years for patients in the primary analysis sample and 50 years for all patients. Among all patients, 90% were HR+, 64% were HER2−, and 54% were pre-menopausal. The mean time since ABC diagnosis was 5.2 years for patients in the primary analysis sample and 6.2 years for all patients. Ten patients (59%) in the primary analysis sample had received (neo)adjuvant therapy (mean 3.7 years since receipt of [neo]adjuvant therapy); among all patients, twenty-eight (57%) had received (neo)adjuvant therapy (mean 4.5 years since receipt of [neo]adjuvant therapy). In the primary analysis sample, 76% of patients were from Ontario compared with 47% among all patients.

Mean age was 47 years for physician respondents and 62% were female. On average, physicians had been in practice for 15.3 years and had treated or managed 80 patients with advanced or metastatic breast cancer in the past year. Eighty-one percent of physicians primarily serve an urban/suburban population. Fifty two percent of physicians were from Ontario, and 9.5% were from each of Quebec and Alberta.

### 3.3. DCE Findings

Estimated patient and physician preference weights for each attribute level are reported in Table 4. It should be noted that these estimates can only be interpreted relative to those of the other levels within a given attribute. The size of the difference between the highest and lowest preference weights within an attribute relative to those for other attributes reflects the importance of that attribute. For example, the effect in the patient primary analysis sample (*n* = 23) of a 53% chance of remaining alive and without cancer progressing (i.e., PFS) over 24 months is 277.0 (134.4–[−142.6] = 277.0), which yields 4.4 times the utility as a noticeable improvement in the amount of pain experienced (31.8–[−31.8] = 63.7). Among all patients (*n* = 62), the effect of a 53% chance of PFS over 24 months was 334.3 (172.1–[−162.2] = 334.3), which yields 5.5 times the amount of utility as a noticeable improvement in the amount of pain experienced (30.4–[−30.4] = 60.8). For physicians, the effect of 53% PFS at 24 months was 386.2 (206.3–[−179.9] = 386.2), or seven times as much utility as noticeable improvement in pain (28.0–[−28.0] = 56.0).

**Figure 2:**
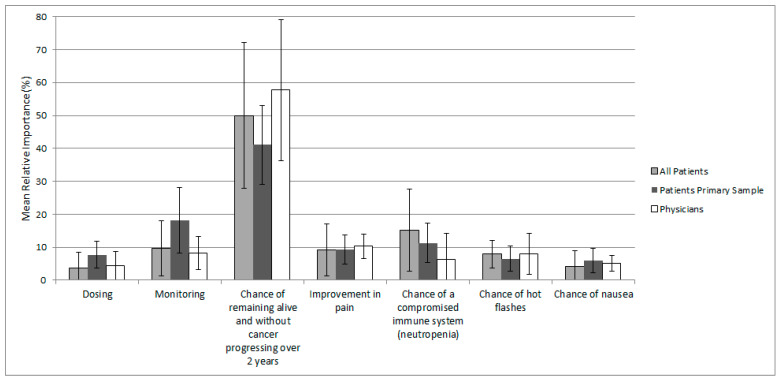
Mean (SD) relative attribute importance for patients and physicians Note: Not all respondents completed every choice task.

The extent to which each attribute influenced treatment choices was generally consistent between patients in the primary analysis sample and among all patients, suggesting that patients would agree on which treatment attributes are most important regardless of menopause, HR, and HER2 statuses (Figure 2). Patients in the primary analysis sample valued the probability of PFS at 24 months as the most important attribute, which was followed by frequency of healthcare services required for follow-up and monitoring, and the chance of a compromised immune system (i.e., neutropenia). For the entire patient sample, the most important attribute was PFS at 24 months, which was followed by the chance of a compromised immune system and frequency of healthcare services required for follow-up and monitoring. Physicians also rated the probability of PFS at 24 months highest, which was followed by improvement in pain and frequency of healthcare services required for follow-up and monitoring.

Preferences weights for different levels of attributes among patients meeting criteria for the primary analysis were generally similar to those for all patients regardless of HR/HER2 and menopausal status. With respect to preference weights for different levels of the dosing regimen attribute, both patients (primary analysis and full sample) and physicians assigned highest preference weights to treatments administered as one tablet daily, corresponding to the dosage regimen for NSAI and TAM. For patients, the estimated preference weights for the levels for the other attributes were generally consistent with expectations, with greater improvements in PFS, noticeable improvement in pain, and lower risks of AEs associated with higher utility values. For physicians, there were instances where results were inconsistent with expectations. For example, on average, physicians assigned a lower utility to bone mineral density tests in addition to blood tests required every 1–3 months than only blood tests required every 1–3 months. Similarly, on average, physicians assigned a lower utility to a 21% chance of nausea than a 29% chance of nausea. These anomalous findings are likely due to the small sample size for physicians.

**Figure 3:**
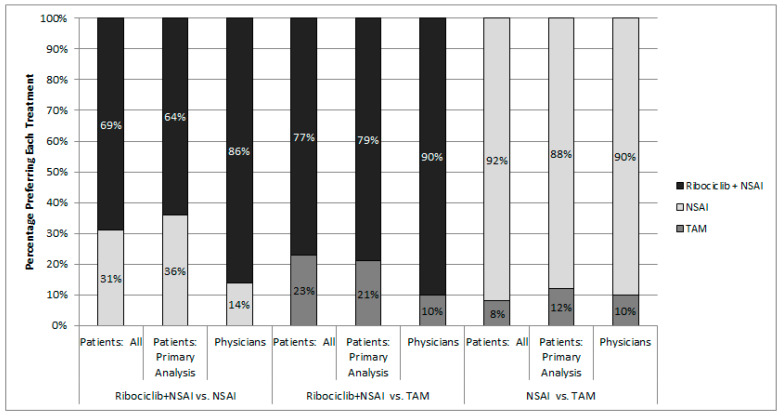
Choice of most preferred treatment in head-to-head comparison tasks; Note: Treatments were not identified by name in the survey.

Results of the head-to-head comparisons of treatments are reported in Figure 3. The treatment with attributes consistent with ribociclib + NSAI was the most frequently selected alternative for both patients and physicians. In the fixed choice tasks comparing treatments corresponding to ribociclib + NSAI versus an NSAI, physicians were more likely than patients to prefer ribociclib + NSAI. Physicians chose ribociclib + NSAI over NSAI 86% of the time compared with 64% for patients in the primary analysis and 69% for all patients. For the fixed-choice task comparing treatments corresponding to ribociclib + NSAI versus TAM, physicians were more likely than patients to prefer with ribociclib + NSAI than TAM. Physicians chose ribociclib + NSAI over TAM 90% of the time compared with 79% for the primary patient analysis and 77% for all patients. Physicians and patients similarly preferred an NSAI to TAM.

The results of head-to-head comparison tasks largely match what would be expected given the results of the attribute-level preference weights. The most weight by far was given to the probability of PFS over 2 years for both patients and physicians. The highest value for this attribute corresponding to actual treatments is 53% for the ribociclib + NSAI treatment option. Therefore, it follows that patients and physicians were more likely to choose ribociclib + NSAI than either NSAI or TAM.

The “fixed” choice tasks were also used to assess transitivity as a validity check. When assessing transitivity, each participant is presented with three choice tasks comparing alternative A vs. B, B vs. C, and A vs. C. Participants who select A over B and B over C should also select A over C. In the primary analysis sample, all patients passed the transitivity test. Among all patients, there were two patients that did not pass the transitivity test. All of the physicians passed the transitivity test. Since such a small number of patients among the full sample failed to adhere to the principle of transitivity, no subgroup analyses were performed among only those who adhered to this principle.

### 3.4. Impact of ABC on Quality of Life and Work and Daily Activities

Tables summarizing the responses to questions regarding patients’ reported feelings of anxiety and depression, perceptions regarding control of their cancer, fear of cancer progression, and effects of ABC on work productivity and daily activities are reported in the Supplementary Materials. Most patients reported experiencing at least some bother from feelings of anxiety, worry, or depression. Most also felt that they or their family or physicians can exert some degree of control over their cancer. A substantial proportion of patients worry about future diagnostic tests and their cancer getting worse. Most reported that cancer had some effect on their ability to work and perform daily activities.

Of the patients who reported that they are currently employed, patients in the primary analysis sample reported that they worked a mean of 23.6 h in the week before taking the survey (21.2 h among all patients). Patients in the primary analysis sample reported that they missed an average of 2.8 h from work in the week before taking the survey because of problems associated with their breast cancer and did not miss any hours of work because of any other reasons (5.1 h among all patients).

## 4. Discussion

### 4.1. Summary

This study assessed patients’ and physicians’ preferences for characteristics of treatments for HR+/HER2− ABC in Canada based on responses to an online survey. The primary analysis focused on pre-/peri-menopausal patients with HR+/HER2− ABC, and a secondary analysis included ABC patients who were postmenopausal, HR-, or HER2+. A DCE was employed, in which respondents were asked to respond to a series of choice tasks in which they were asked to choose between two hypothetical or actual treatment alternatives. Each treatment alternative was characterized by seven attributes related to dosing regimen, efficacy profile, and risks of AEs, with levels of these attributes corresponding approximately to those for with ribociclib + NSAI, an NSAI, and TAM based on the MONALEESA-7 trial and other sources. For patients, the impacts of ABC on QOL and ability to work and perform activities of daily living (ADL) also were assessed.

Rankings of treatment attributes in terms of their importance were similar for patients in the primary analysis sample compared with all patients. For patients, the most important treatment attribute was the chance of PFS at 24 months, which was followed by the frequency of healthcare services required for follow-up and monitoring, and the chance of a compromised immune system (i.e., neutropenia). For physicians, the most important attribute was the probability of PFS at 24 months, which was followed by improvement in pain, and the frequency of healthcare services required for follow-up and monitoring. With respect to preference weights for different levels for the dosing regimen, patients and physicians assigned highest preference weights to regimens that require one tablet daily by mouth, corresponding to NSAI and TAM. Across all fixed choice tasks, patients chose a treatment with attributes consistent with ribociclib + NSAI 70% of the time, while physicians chose it 88% of the time. Patients and physicians chose ribociclib + NSAI 64% and 86% of the time over an NSAI and 79% and 90% over TAM, respectively. Most patients reported being bothered by feelings of anxiety, worry, or depression; that they worry about future diagnostic tests, about their cancer getting worse, and that ABC has some impact on their ability to work or perform daily activities.

### 4.2. Comparison with Prior Studies

To the best of our knowledge, few studies have been published that assessed preferences of pre-/peri-menopausal women with HR+/HER2− ABC or physicians who treat such patients for treatment attributes corresponding to endocrine-based therapies. One study involving patients with metastatic breast cancer and nurses and oncologists who treat such patients evaluated preferences for attributes for OS in addition to PFS and risks of severe AEs [40]. However, the authors note that preferences for PFS and OS were assessed with a single attribute represented by a sequence of varying health states for stable and progressive disease. This allowed the investigators to measure preferences for prolonged PFS *while holding OS constant*, but it was not designed to assess whether respondents placed higher value on prolonged OS compared with prolonged PFS. Results suggested that patients prefer longer periods of PFS regardless of whether there is a prolongation of OS when choosing among treatments; however, oncologists’ and nurses’ preferences were not influenced by prolonged PFS with no prolonged OS [40]. In this same study, patients, oncologists, and nurses preferred lower chances of experiencing AEs [40]. The study reported here did not include an attribute for OS, because these data from MONALEESA-7 remained immature and had not been published prior to the conduct of this study; nevertheless, results are otherwise consistent with the aforementioned study by MacEwan and colleagues in that patients most valued greater probability of PFS among all attributes. Another study that evaluated preferences of postmenopausal patients with HR+/HER2− ABC for treatment attributes corresponding to an AI with or without mechanistic target of rapamycin (MTOR) inhibitor in Thailand reported that such patients prefer treatments with greater PFS and lower chance of experiencing side effects [18]. These findings are consistent with the study reported here, which would indicate that HR+/HER2− breast cancer patients have similar preferences regardless of menopausal status. Other studies that evaluated preferences of breast cancer patients for attributes of CTs indicated that patients placed highest importance on reduction of risks of AEs [41,42]. Unlike the study here, the study by Beusterien and colleagues only included attributes for risks of side effects associated with CT [36]. The study by Spaich and colleagues did include attributes for PFS but found that this ranked lower than decreased risks of experiencing neutropenia, alopecia, and neuropathy [35]. These differences may be due to the fact that the studies by Beusterien et al. and Spaich et al. included treatments for CT regimens only, whereas this study focused on ET [41,42].

### 4.3. Limitations

The main limitation of this study was the relatively small sample size, especially for the physician sample. In addition, many patients failed to complete all the choice tasks, which was possibly due to difficulties in understanding the questions or the length of the survey. Responses from patients who completed the pre-test (*n* = 2) indicated that the instructions for completing the survey, the descriptions of the characteristics of the treatments, and the choice task questions were easy to understand. Of the 118 patients who started the survey, only 27 met the criteria for the primary analysis. Still, subgroup analyses of all patients regardless of menopause, HR, and HER2 status revealed that such patients placed the highest importance on the probability of PFS, which is similar to pre-/peri-menopausal patients with HR+/HER2− ABC. This would indicate that preferences among patients in the primary analysis and among all patients are generally consistent. Importantly, mature data on OS for the treatments of interest in pre-/peri-menopausal patients with HR+/HER2− ABC were not available at the time this study was conducted, and as such, OS was not included as an attribute in the DCE. Long considered the gold standard as evidence of efficacy in randomized controlled trials (RCT), data on OS are often unavailable from the preliminary analyses of trials of novel treatments for breast cancer. Patients were recruited by email and from social media sites by a Canadian patient advocacy group. Patients enrolled through these channels may not be representative of all ABC patients in Canada. Similarly, physicians were recruited based on engagement with Novartis, and only 21 of 271 who were recruited (8%) completed at least one DCE question; hence, they may not be representative of all physicians treating ABC patients in Canada.

## 5. Conclusions

Given the low completion rate for patients (19%) and the low response rate among physicians recruited for the survey (8%), the findings of this study must be interpreted cautiously. Limitations notwithstanding, this study suggests that patients and physicians may prefer treatments with attributes consistent with those of ribociclib plus NSAI in combination over those with attributes consistent with those of NSAI or TAM. Thus, access to treatment with CDK4/6 inhibitors—such as ribociclib—provides an opportunity to improve satisfaction of patients with ABC in Canada. It is important that these findings be considered by policy makers in their deliberations regarding reimbursement and access to novel treatments for pre-/peri-menopausal patients with HR+/HER2− ABC.

## Figures and Tables

**Table 1:** Attributes and levels included in the discrete choice experiment (DCE).

Attribute	Level 1	Level 2	Level 3
Dosing regimen	One tablet daily	Two medications: For first medication, three tablets daily for 21 consecutive days followed by 7 days off treatment; For second medication, one tablet daily	--
Monitoring	No electrocardiograms or bone mineral density tests; and blood tests every 1 to 3 months	Bone mineral density tests every 3 months; and blood tests every 1 to 3 months	Electrocardiograms every 2 weeks for 6 months, then every 3 months; bone mineral density tests every 3 months; and blood tests every 2 weeks for the first 2 months, then once every month for 4 months, and every 1 to 3 months thereafter
Chance of remaining alive and progression-free over 24 months	25%	35%	53%
Amount of pain experienced	No noticeable improvement	Noticeable improvement	--
Chance of neutropenia	7%	78%	--
Chance of hot flashes	21%	24%	29%
Chance of nausea	20%	31%	--

Note: The levels in this table do not correspond to any particular treatment.

**Table 2:** Attributes profiles for treatments of advanced breast cancer.

Attribute	Ribociclib + NSAI	NSAI	Tamoxifen
Dosing schedule	Two medications: For first medication, three tablets daily for 21 consecutive days followed by 7 days off treatment; For second medication, one tablet daily	One tablet daily	One tablet daily
Monitoring	Electrocardiograms every 2 weeks for 6 months, then every 3 months; bone mineral density tests every 3 months; and blood tests every 2 weeks for the first 2 months, then once every month for 4 months, and every 1 to 3 months thereafter	Bone mineral density tests every 3 months; and blood tests every 1 to 3 months	No electrocardiograms or bone mineral density tests; and blood tests every 1 to 3 months
Chance of remaining alive and progression-free over 24 months	53%	35%	25%
Amount of pain experienced	Noticeable improvement	No noticeable improvement	No noticeable improvement
Chance of neutropenia	78%	7%	7%
Chance of hot flashes	21%	24%	29%
Chance of nausea	31%	20%	20%

Sources [16,26,27,28,29,30,31,32].

**Table 3:** Demographic characteristics: patients (all and primary) and physicians.

Characteristic	Primary Analysis (*n* = 27)	All Patients (*n* = 118)	Physicians (*n* = 21)
Age, years, Mean (SD) *	46.1 (9.3)	50.0 (8.6)	46.6 (7.8)
Gender, *n* (%)			
Female	27 (100)	115 (100)	13 (61.9)
Province of Residence, *n* (%)			
Alberta	2 (11.8)	9 (18.4)	2 (9.5)
British Columbia	1 (5.9)	7 (14.3)	4 (19.0)
New Brunswick	0	3 (6.1)	0
Newfoundland and Labrador	0	2 (4.1)	1 (4.8)
Nova Scotia	0	0	1 (4.8)
Ontario	13 (76.5)	23 (46.9)	11 (52.4)
Prince Edward Island	0	1 (2.0)	0
Quebec	0	0	2 (9.5)
Saskatchewan	1 (5.9)	4 (8.2)	0
Highest education level attained, *n* (%)			
High school	2 (11.8)	9 (18.4)	---
Associates degree	5 (29.4)	14 (28.6)	---
Bachelor’s degree	4 (23.5)	14 (28.6)	---
Postgraduate degree	6 (35.3)	12 (24.5)	---
Employment (work for pay), *n* (%)			
Full-time	2 (11.8)	9 (18.4)	---
Part-time	6 (35.3)	9 (18.4)	---
Unemployed	9 (52.9)	31 (63.3)	---
HR status			
HR+	27 (100)	79 (89.8)	---
HR-	0	9 (10.2)	---
HER2 status			
HER2+	0	30 (35.7)	---
HER2−	27 (100)	54 (64.3)	---
Pre-menopausal			
Yes	27 (100)	57 (54.3)	---
No	0	48 (45.7)	---
Stage of ABC when diagnosed			
Stage I or II	5 (29.4)	14 (28.6)	---
Stage III	5 (29.4)	17 (34.7)	---
Stage IV	7 (41.2)	18 (33.7)	---
Taking medication for ABC, *n* (%)			
Currently	15 (88.2)	42 (85.7)	---
Not currently but did previously	1 (5.9)	2 (4.1)	---
Never	1 (5.9)	5 (10.2)	---
Medications currently or previously taken/received, *n* (%)			
Ribociclib (Kisqali)	3 (4.5)	4 (2.1)	---
Palbociclib (Ibrance)	7 (10.6)	18 (9.6)	---
Tamoxifen	12 (18.2)	25 (13.4)	---
Letrozole (Femara)	11 (16.7)	30 (16.0)	---
Anastrozole (Arimidex)	2 (3.0)	8 (4.3)	---
Fulvestrant (Faslodex)	5 (7.6)	12 (6.4)	---
Exemestane (Aromasin)	3 (4.5)	11 (5.9)	---
Unknown (e.g., due to trial participation)	0	2 (1.1)	---
Chemotherapy	10 (15.2)	31 (16.6)	
Other	8 (12.1)	19 (10.2)	---
Time since most recent physician visit for management of ABC, *n* (%)			
Less than one month	13 (76.5)	38 (77.6)	---
One to three months	2 (11.8)	6 (12.2)	---
Three to six months	0	3 (6.1)	---
Six to 12 months	2 (11.8)	2 (4.1)	---
Specialty of physician seen most regularly for management of ABC, *n* (%)			
Medical Oncologist	16 (94.1)	45 (91.8)	---
Primary Care	0	1 (2.0)	---
Other	1 (5.9)	3 (6.1)	---
Work setting			
Community-based health center	---	---	8 (38.1)
Academic health center	---	---	13 (61.9)

* Mean age calculated based on midpoints within age categories.

**Table 4:** Attribute-level preference weights.

Attribute Level	Primary Analysis Patients (*n* = 23) Mean (SD)	All Patients (*n* = 62) Mean (SD)	Physicians (*n* = 21) Mean (SD)
Dosing regimen			
Two medications: For first medication, three tablets daily for 21 consecutive days followed by 7 days off treatment; for second medication, one tablet daily	−17.2 (26)	−1.3 (21)	−1.1 (22)
One tablet daily	17.2 (26)	1.3 (21)	1.1 (22)
Monitoring			
Electrocardiograms every 2 weeks for 6 months, then every 3 months; bone mineral density tests every 3 months; and blood tests every 2 weeks for the first 2 months, then once every month for 4 months, and every 1 to 3 months thereafter	−74.8 (42)	−29.4 (40)	−19.0 (26)
Bone mineral density tests every 3 months; and blood tests every 1 to 3 months	29.7 (26)	16.5 (18)	28.2 (18)
Blood tests every 1 to 3 months	45.1 (32)	12.9 (34)	−9.2 (18)
Chance of remaining alive and without cancer progressing over 2 years			
53%	134.4 (65)	172.1 (101)	206.3 (80)
35%	8.2 (38)	−9.9 (32)	−26.4 (30)
25%	−142.6 (37)	−162.2 (85)	−179.9 (107)
Improvement in pain			
Noticeable improvement	31.8 (16)	30.4 (29)	28.0 (27)
No noticeable improvement	−31.8 (16)	−30.4 (29)	−28.0 (27)
Chance of a compromised immune system (neutropenia)			
7%	39.0 (22)	50.5 (47)	15.3 (32)
78%	−39.0 (22)	−50.5 (47)	−15.3 (32)
Chance of hot flashes			
21%	−1.3 (16)	−7.3 (27)	−21.4 (31)
24%	−0.3 (25)	−7.8 (16)	7.3 (30)
29%	1.6 (26)	15.1 (29)	14.1 (10)
Chance of nausea			
31%	−10.1 (22)	−9.3 (20)	−4.3 (20)
20%	10.1 (22)	9.3 (20)	4.3 (20)

Note: Not all respondents completed every choice task.

## Supplementary Materials

The following are available online at https://www.mdpi.com/1718-7729/28/1/51/s1, Table S1: Patients’ reported feelings of anxiety and depression; Table S2: Patients’ perceptions regarding control of their cancer; Table S3: Patients fear of cancer progression; Table S4: Patients’ perceptions regarding the effects of their cancer on fertility; Supplementary Materials 1: Survey instrument.
