# Supplementary material for: Preferences of Canadian Patients and Physicians for Treatment of HR+/HER2− Advanced Breast Cancer
Source: Curr Oncol. 2021 Jan 14;28(1):491–508. doi: 10.3390/curroncol28010051 (PMC7903278; doi:10.3390/curroncol28010051)
Supplement: Supplementary file 1 [file curroncol-28-00051-s001.pdf]

Copyright © Multimed Inc.

Supplemental Materials for

Preferences of Canadian Patients and Physicians for Treatment of HER2-/HR+ Advanced Breast Cancer

Stellato D, BS; Thabane M, PhD; Eichten C, BA; Delea TE, MSIA

Listing of Supplemental Material(s):

Supplemental Table 1: Patients' reported feelings of anxiety and depression

Supplemental Table 2: Patients' perceptions regarding control of their cancer

Supplemental Table 3: Patients fear of cancer progression

Supplemental Table 4: Patients' perceptions regarding the effects of their cancer on fertility

Supplemental Appendix 1: Survey instrument

Copyright © Multimed Inc.

Supplemental Table 1. Patients' reported feelings of anxiety and depression

	Frequency That Patients Were Bothered by These Problems Over Past 2 Weeks, N (%)			
	Not at all	Fewer than half of the days	More than half of the days	Nearly every day
**Primary Analysis (N=17)**				
Feeling nervous, anxious, or on edge	4 (23.5)	6 (35.3)	6 (35.3)	1 (5.9)
Not being able to stop or control worrying	5 (29.4)	8 (47.1)	4 (23.5)	0
Feeling down, depressed, or hopeless	5 (29.4)	7 (41.2)	5 (29.4)	0
Little interest or pleasure in doing things	5 (29.4)	7 (41.2)	5 (29.4)	0
**All Patients (N=48)**				
Feeling nervous, anxious, or on edge	12 (24.5)	22 (44.9)	12 (24.5)	3 (6.1)
Not being able to stop or control worrying	19 (38.8)	19 (38.8)	8 (16.3)	3 (6.1)
Feeling down, depressed, or hopeless	21 (42.9)	18 (36.7)	8 (16.3)	2 (4.1)
Little interest or pleasure in doing things	22 (44.9)	17 (34.7)	9 (18.4)	1 (2.0)

Supplemental Table 2. Patients' perceptions regarding control of their cancer

	Extent to which Patients Agree with Statements Regarding Their Cancer, N (%)				
	Strongly Disagree	Disagree	Neither Agree nor Disagree	Agree	Strongly Agree
**Primary Analysis (N=17)**					
I can definitely influence the course of the cancer	0	2 (11.8)	6 (35.3)	8 (47.1)	1 (5.9)
My doctor can definitely influence the course of the cancer	0	1 (5.9)	4 (23.5)	8 (47.1)	4 (23.5)
The key people in my life can definitely influence the course of the cancer	2 (11.8)	2 (11.8)	6 (35.3)	5 (29.4)	2 (11.8)

Copyright © Multimed Inc.

By taking extra care of myself I can influence the course of the cancer	0	1 (5.9)	7 (41.2)	7 (41.2)	2 (11.8)
By living healthily I can influence the course of the cancer.	0	1 (5.9)	7 (41.2)	7 (41.2)	2 (11.8)
If I follow the advice of my doctor, I can definitely influence the course of the cancer	0	0	4 (23.5)	12 (70.6)	1 (5.9)
I can influence the course of the cancer by fighting against it.	1 (5.9)	3 (17.6)	4 (23.5)	6 (35.3)	3 (17.6)
**All Patients (N=48)**					
I can definitely influence the course of the cancer	1 (2.1)	5 (10.4)	12 (25.0)	24 (50.0)	6 (12.5)
My doctor can definitely influence the course of the cancer	0	3 (6.3)	7 (14.6)	23 (47.9)	15 (31.3)
The key people in my life can definitely influence the course of the cancer	3 (6.3)	7 (14.6)	14 (29.2)	16 (33.3)	8 (16.7)
By taking extra care of myself I can influence the course of the cancer	0	4 (8.3)	12 (25.0)	22 (45.8)	10 (20.8)
By living healthily I can influence the course of the cancer.	1(2.1)	3 (6.3)	11 (22.9)	25 (52.1)	8 (16.7)
If I follow the advice of my doctor, I can definitely influence the course of the cancer	0	1 (2.1)	10 (20.8)	26 (54.2)	11 (22.9)
I can influence the course of the cancer by fighting against it.	3 (6.3)	7 (14.6)	10 (20.8)	14 (29.2)	14 (29.2)

Supplemental Table 3. Patients fear of cancer progression

	Extent to Which Patients Worry about Future Diagnostic Tests, Other Types of Cancer, and Their Cancer Getting Worse, N (%)			
	Not at All	A little bit	Somewhat	Very much

Copyright © Multimed Inc.

**Primary Analysis (N=17)**				
Future diagnostic tests	2 (11.8)	4 (23.5)	5 (29.4)	6 (35.3)
Another type of cancer	6 (35.3)	6 (35.3)	2 (11.8)	3 (17.6)
My cancer getting worse	1 (5.9)	1 (5.9)	2 (11.8)	13 (76.5)
**All Patients (N=48)**				
Future diagnostic tests	10 (20.8)	10 (20.8)	18 (37.5)	10 (20.8)
Another type of cancer	13 (27.1)	15 (31.3)	12 (25.0)	8 (16.7)
My cancer getting worse	2 (4.2)	3 (6.3)	11 (22.9)	32 (66.7)

Supplemental Table 4. Patients' perceptions regarding the effects of their cancer on fertility

	Extent to which Patients Agree with Statements Regarding Their Cancer, N (%)				
	Strongly Disagree	Disagree	Neither Agree nor Disagree	Agree	Strongly Agree
**Primary Analysis (N=17)**					
The thought of telling my (potential) spouse/partner that I may be unable to have children makes me uncomfortable.	6 (35.3)	2 (11.8)	8 (47.1)	1 (5.9)	0
I am worried about passing on a genetic risk for cancer to my children.	1 (5.9)	0	1 (5.9)	8 (47.1)	7 (41.2)
I am cautious about having (more) children because I might not be around to raise them.	3 (17.6)	3 (17.6)	5 (29.4)	2 (11.8)	4 (23.5)
I will be happy with life whether or not I have (more) children someday.	1 (5.9)	1 (5.9)	6 (35.3)	7 (41.2)	2 (11.8)
**All Patients (N=48)**					
The thought of telling my (potential) spouse/partner that I may be unable to	16 (32.7)	6 (12.2)	25 (51.0)	2 (4.1)	0

Copyright © Multimed Inc.

have children makes me uncomfortable.					
I am worried about passing on a genetic risk for cancer to my children.	4 (8.2)	3 (6.1)	8 (16.3)	18 (36.7)	16 (32.7)
I am cautious about having (more) children because I might not be around to raise them.	7 (14.3)	3 (6.1)	27 (55.1)	2 (4.1)	10 (20.4)
I will be happy with life whether or not I have (more) children someday.	5 (10.2)	1 (2.0)	21 (42.9)	12 (24.5)	10 (20.4)

Supplemental Appendix 1. Survey instrument

Acrobat Document
